# Effect of renal function on the diagnostic performance of plasma biomarkers for Alzheimer’s disease

**DOI:** 10.3389/fnagi.2023.1150510

**Published:** 2023-03-15

**Authors:** Bin Zhang, Cheng Zhang, YuYe Wang, LeiAn Chen, YaNan Qiao, Yu Wang, DanTao Peng, for the Alzheimer’s Disease Neuroimaging Initiative

**Affiliations:** ^1^Department of Neurology, China-Japan Friendship Hospital, Chinese Academy of Medical Sciences and Peking Union Medical College, Beijing, China; ^2^School of Acupuncture, Moxibustion and Tuina, International Acupuncture and Moxibustion Innovation Institute, Beijing University of Chinese Medicine, Beijing, China; ^3^Department of Neurology, China-Japan Friendship Hospital, Beijing, China

**Keywords:** Alzheimer’s disease, plasma biomarkers, renal function, diagnostic performance, cutoff

## Abstract

**Background:**

Several blood-based biomarkers are promising to be used in the diagnosis of Alzheimer’s disease (AD) including Aβ42/40, p-tau181, and neurofilament light (NfL). The kidney is associated with the clearance of proteins. It is crucial to evaluate the effect of renal function on the diagnostic performance of these biomarkers before clinical implementation, which is important for the establishment of reference ranges and the interpretation of results.

**Methods:**

This study is a cross-sectional analysis based on ADNI cohort. Renal function was determined by the estimated glomerular filtration rate (eGFR). Plasma Aβ42/40 was measured by liquid chromatography–tandem mass spectrometry (LC–MS/MS). Plasma p-tau181 and NfL were analyzed by Single Molecule array (Simoa) technique. [18F] florbetapir-PET (Aβ-PET) was used as a reference standard to estimate the brain amyloid load. The cutoff of Aβ-PET positivity was defined as ≥1.11. Linear regression models were used to investigate the associations of continuous eGFR with each plasma biomarker separately. The diagnostic accuracies of plasma biomarkers for positive brain amyloid across different renal function groups were analyzed by Receiver operating characteristic (ROC) curve. Youden-Index was used to determine the cutoff levels.

**Results:**

A total of 645 participants were included in this study. The levels and diagnostic performance of Aβ42/40 were not affected by renal function. eGFR was only found negatively associated with p-tau181 levels in Aβ-PET negetive sample (*β* = −0.09, *p* = 0.039). eGFR was found negatively associated with NfL levels both in whole sample and Aβ-PET stratified groups (*β* = −0.27, *p* < 0.001 in whole sample; *β* = −0.28, *p* = 0.004 in A^−^; *β* = −0.27, *p* < 0.001 in A^+^). The diagnostic accuracies of p-tau181 and NfL were not affected by renal function. But the cutoff values of p-tau181 and NfL changed in participants with mild to moderate eGFR decline compared to participants with normal eGFR.

**Conclusion:**

Plasma Aβ42/40 was a robust biomarker for AD which was not affected by renal function. Plasma p-tau181 and NfL levels were affected by renal function, specific reference values of them should be considered in populations with different renal function stages.

## 1. Introduction

Alzheimer’s disease (AD) is a progressive neurodegenerative disease that is the most common form of dementia. The estimated total number of persons across the AD continuum including AD dementia, prodromal AD, and preclinical AD was 416 million (Gustavsson et al., 2022). The exact etiology of AD has not been elucidated. Classical pathogenesis hypotheses of AD include β-amyloid (Aβ) deposition and neurofibrillary tangles (NFT) due to the accumulation of hyperphosphorylated tau in the brain. Clinical criteria are the primary foundation for the diagnosis of AD (McKhann et al., 2011). However, the pathological changes of AD occur several years before the appearance of clinical symptoms (Jack et al., 2013). In 2018, to achieve the early detection of AD pathology, the National Institute on Aging-Alzheimer’s Association (NIA-AA) Research Framework created a biological definition of AD (Jack et al., 2018). The specific biomarkers of AD are classified into Aβ deposition, pathologic tau, and neurodegeneration [ATN], which can be examined by cerebrospinal fluid (CSF) and neuroimaging. However, these examinations are invasive or expensive and are difficult to be implemented in general clinical settings. Blood biomarkers for AD are becoming increasingly important due to their non-invasive and cheap characteristics.

With the development of highly sensitive technologies, several blood-based biomarkers for AD have shown good diagnostic value in different cohorts, among which Aβ42/40, p-tau181, and Neurofilament Light (NfL) are promising for clinical applications (Hansson et al., 2022; Teunissen et al., 2022). Before blood-based biomarkers are used in the clinical settings, further clarification of which factors affect their levels is needed, which is important for the development of reference ranges and the interpretation of results. The kidney is the most important excretory organ of the body, and some studies have reported that chronic kidney disease (CKD) can lead to elevated plasma levels of Aβ42, Aβ40, p-tau181 and NfL (Mielke et al., 2022; Syrjanen et al., 2022), which may due to the decreased renal clearance (Cheng et al., 2020). However, the effect of renal function on the diagnostic performance of plasma Aβ42/40 and NfL has not been investigated so far. Besides, CKD is a severe renal dysfunction, whether mild to moderate renal function decline affects the levels of blood biomarkers for AD need to be determined. Against this background, this study aimed to investigate the effect of renal function across three stages on the diagnostic performance of plasma Aβ42/40, p-tau181, and NfL for AD.

## 2. Methods

### 2.1. Participants

We used data from the Alzheimer’s Disease Neuroimaging Initiative (ADNI) database (adni.loni.usc.edu, accessed on 4th December 2022). ADNI is a non-randomized cohort study launched in North America in 2003 (Mueller et al., 2005). The major goal of ADNI is to develop biomarkers for the early diagnosis of AD. The participants in ADNI include cognitively normal (CN), significant memory concern (SMC), early MCI (EMCI), late MCI (LMCI), and mild AD. Detailed inclusion and exclusion criteria of participants can be found on the ADNI website. Briefly, all enrolled subjects must be 55–90 years old with a good command of English or Spanish. All subjects will undergo a series of tests including clinical and neuropsychological assessment, neuroimaging, and biomarker measurement. Participants with any significant neurologic disease other than AD, severe psychiatric disorders, or significant systemic illness will be excluded. The ADNI study was authorized by the regional ethics council, and all participants provided written informed consent. Our study is a cross-sectional analysis based on ADNI cohort. We included all ADNI participants with serum creatinine test and Aβ-PET examination and at least one plasma biomarker measurement at baseline.

### 2.2. Assessment of renal function

Non-fasting serum creatinine was quantified on Roche/Hitachi cobas c systems. The estimated glomerular filtration rate (eGFR) was calculated according to the new Chronic Kidney Disease Epidemiology Collaboration (CKD-EPI) equation (Inker et al., 2021). New eGFR equations use serum creatinine incorporate age and sex without race to evaluate renal function. Participants were categorized by eGFR into the following groups; eGFR <60 ml/min/1.73 m^2^ was defined as moderate renal function decline, eGFR 60–90 ml/min/1.73 m^2^ was defined as mild renal function decline, and eGFR ≥90 ml/min/1.73 m^2^ was defined as normal renal function. Besides, we searched the medical record in ADNI database by using CKD, acute kidney injury, kidney failure, and nephrectomize as search terms. No participants included in our analysis had these severe renal dysfunctions at baseline.

### 2.3. Measures of plasma biomarkers

Morning fasting plasma samples were collected into EDTA plastic tubes and were stored at −80°C before sample analysis. Plasma Aβ42/40 was measured by a high-precision liquid chromatography–tandem mass spectrometry (LC–MS/MS) as previously described (Ovod et al., 2017). Plasma p-tau181 and NfL were analyzed by validated ultrasensitive Single Molecule array (Simoa) technique, at the Clinical Neurochemistry Laboratory, University of Gothenburg, Sweden (Mattsson et al., 2019; Karikari et al., 2021). The lower limit of quantification (LOQ) was 1.0 pg./ml for p-tau181 and 6.7 pg./ml for NfL. Scientists who measured the plasma biomarkers were blinded to participants’ clinical information. Only values that passed quality control were used for further data analyses.

### 2.4. Amyloid PET imaging

[18F] florbetapir-PET (Aβ-PET) was used as a reference standard to estimate the brain Aβ load. The summary SUVR of frontal, anterior/posterior cingulate, lateral parietal, and lateral temporal regions were generated using the whole cerebellum as a reference region. Aβ-PET positivity was defined according to the cutoff of 1.11 (Landau et al., 2012, 2013).

### 2.5. Statistical analyses

The normality of all continuous data was detected by Shapiro–Wilk test. Normally distributed continuous data was presented as the mean ± standard deviation and compared by one-way analysis of variance (ANOVA). Skewed distributed continuous data was presented as the median and interquartile range (IQR) and compared by Kruskal Wallis non-parametric test. Categorical data was presented as the number (%) and compared by Pearson chi-square test.

The associations of continuous eGFR with each plasma biomarker separately were investigated by linear regression, including age and sex as covariates. Then we performed these analyses stratified by Aβ-PET status (A^−^ represents Aβ-PET negative, A^+^ represents Aβ-PET positive) to evaluate if the associations between eGFR and plasma biomarkers were modified by Alzheimer’s pathology.

The diagnostic accuracies of plasma biomarkers for positive Aβ-PET across different eGFR categories were analyzed by Receiver operating characteristic (ROC) curve. Youden-Index was used to determine the cutoff levels, and the sensitivity, specificity, positive predictive value (PPV), and negative predictive value (NPV) were also calculated. Differences between the area under the ROC curve (AUC) compared with the lowest eGFR categories (eGFR <60) were evaluated by the DeLong test (DeLong et al., 1988). We also estimated the eGFR-adjusted AUC for each plasma biomarker.

All statistical analyses were conducted using the SPSS statistical software (version, 26.0, SPSS Inc., Chicago, IL, USA), MedCalc software (version 20.022, MedCalc, Ostend, Belgium), and the *ROCt* package of R software (version 4.1.2). Two-sided *p* < 0.05 was considered statistically significant for all tests.

## 3. Results

### 3.1. Participant characteristics

**Figure 1:**
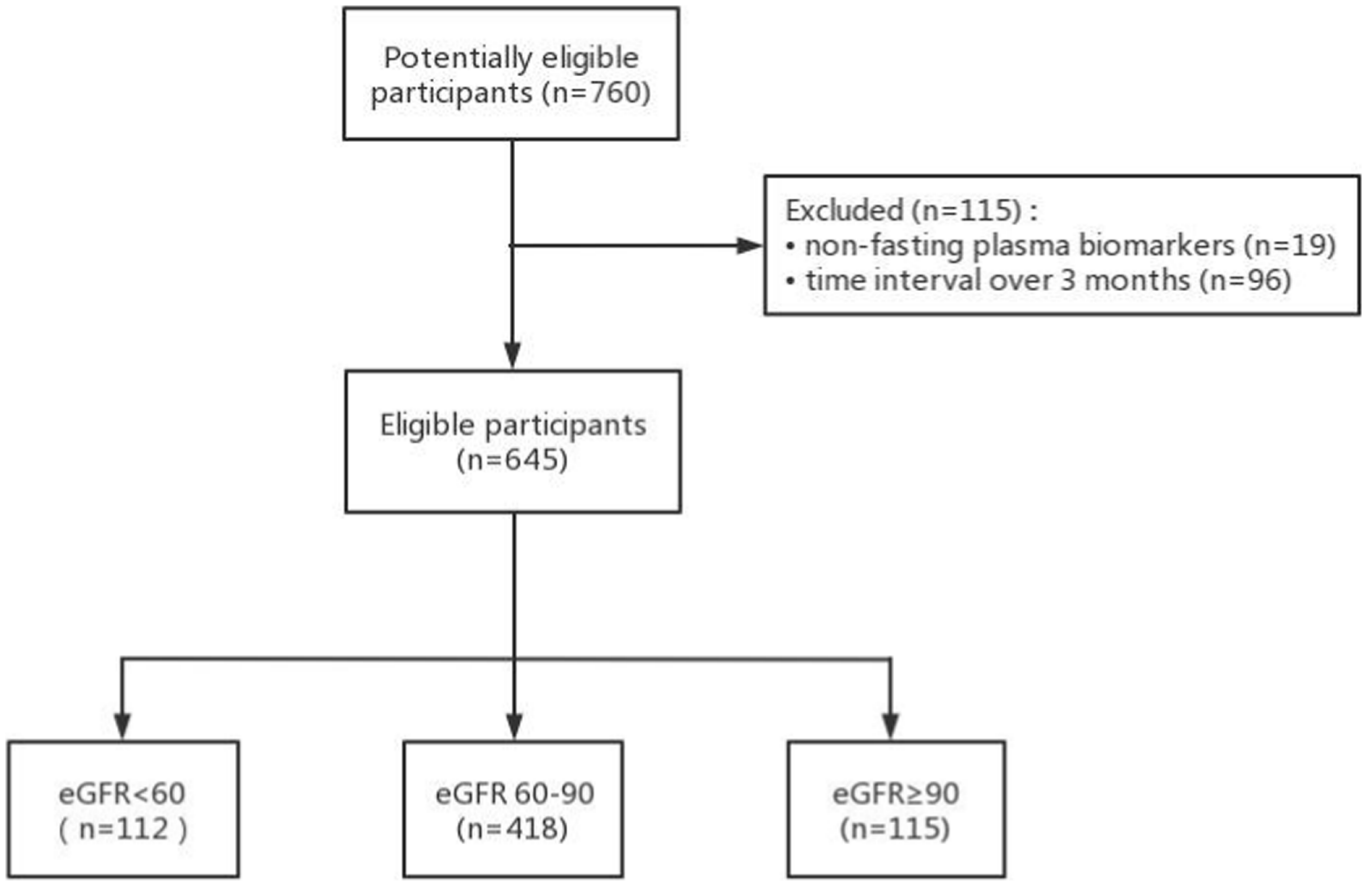
Flow diagram.

A total of 760 participants had creatinine, Aβ-PET data, and at least one plasma biomarker examination (Figure 1.). 19 participants with non-fasting plasma and 96 participants with the time intervals between serum creatinine, plasma biomarkers, and Aβ-PET examination over 90 days were excluded. Finally, we included 645 participants in this study comprising 333 participants with Aβ42/40, 639 participants with p-tau181, and 638 participants with NfL measurements. The mean interval between the plasma collection and the Aβ-PET scan was 3.5 days with a range of 0–78 days. The mean interval between the serum creatinine examination and the amyloid PET scan was 42.5 days with a range of 0–89 days. The mean interval between the serum creatinine examination and the plasma collection was 39.0 days with a range of 0–89 days.

**Figure 2:**
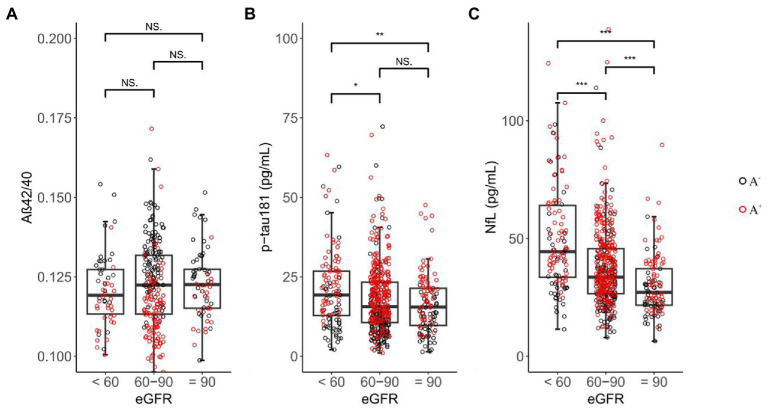
Inter-group differences in plasma biomarkers among eGFR groups. NS = not significant, 0.01 ≤ * ≤ 0.05, 0.001 ≤ ** < 0.01, *** < 0.001; black circle represents Aβ-PET negative sample, red circle represents Aβ-PET positive sample.

Baseline demographic characteristics stratified by eGFR categories are presented in Table 1. As expected, participants with a lower eGFR were older (median age = 77.95 for eGFR <60, 72.05 for eGFR 60–90, 70.00 for eGFR ≥90 ml/min, *p* < 0.001). Baseline p-tau 181 concentrations were significantly higher in eGFR <60 (median = 19.25 pg./ml) than eGFR 60–90 (median = 15.60 pg./ml; *p* = 0.014; Figure 2) and eGFR ≥90 ml/min (median = 15.45 pg./ml; *p* = 0.004). For eGFR 60–90 versus eGFR ≥90 ml/min, levels of p-tau 181 did not differ (*p* = 0.271). Within each eGFR group, levels of NfL were significantly higher among those with lower eGFR than among those with higher eGFR (median levels for an eGFR <60, 60–90, and ≥ 90 ml/min were 44.45, 33.60, and 27.15 pg./ml, respectively, *p* < 0.001). No significant difference was found between each eGFR group for Aβ42/40 (*p* = 0.461).

**Table 1:** Demographics of participants^a^.

	eGFR < 60 (*n* = 112)	eGFR 60–90 (*n* = 418)	eGFR ≥ 90 (*n* = 115)	*P*
Age	77.95 (9.87)	72.05(9.80)	70.00 (6.85)	<0.001
Sex:Female, No.(%)	57 (50.9)	194 (46.4)	50 (43.5)	0.526
Education (years)	16.50 (5.00)	16.00 (4.00)	16.00 (4.00)	0.37
≥1 *APOE* ε4, No.(%)	44 (39.3)	195 (46.6)	60 (52.2)	0.147
Diagnose, No.(%) CN	38 (33.9)	89 (21.3)	25 (21.7)	0.12
SMC	1 (0.9)	13 (3.1)	2 (1.7)	
EMCI	32 (28.6)	160 (38.3)	50 (43.5)	
LMCI	24 (21.4)	85 (20.3)	23 (20.0)	
AD	17 (15.2)	71 (17.0)	15 (13.0)	
Creatinine (mg/dL)	1.30 (0.30)	1.00 (0.20)	0.80 (0.20)	<0.001
eGFR	53.38 (9.61)	72.59 (12.20)	93.80 (4.48)	<0.001
Aβ-PET positive, No.(%)	61 (54.5)	228 (54.5)	58 (50.4)	0.727
Plasma Aβ42/40	0.12 (0.02)	0.12 (0.02)	0.12 (0.01)	0.461
p-tau181 (pg/mL)	19.25 (14.00)	15.60 (12.64)	15.45 (11.64)	0.012
NfL (pg/mL)	44.45 (30.45)	33.60 (19.14)	27.15 (15.48)	<0.001

^a^Data are presented as the mean ± standard deviation for normally distributed variables, median and interquartile range (IQR) for skewed distributed variables, and number (%) for categorical variables.

### 3.2. Association between renal function and plasma biomarkers

As shown in Table 2, eGFR was not associated with Aβ42/40 ratio whether in whole sample or Aβ-PET stratified groups. eGFR was only found negatively correlated with p-tau181 levels in A^−^ (*β* = −0.09, *p* = 0.039; adjusted model). eGFR was found negatively associated with NfL levels both in whole sample and Aβ-PET stratified groups (*β* = −0.27, *p* < 0.001 in whole sample; *β* = −0.28, *p* = 0.004 in A^−^; *β* = −0.27, *p* < 0.001 in A^+^; adjusted model). No interaction effects were found between Aβ-PET status and eGFR for each plasma biomarker in adjusted model (*p* for interaction were 0.921, 0.988, and 0.990 for Aβ42/40, p-tau181, and NfL, respectively).

**Table 2:** Cross-sectional association between eGFR and plasma biomarkers^a^.

	*N*	Unadjusted model			Adjusted model^b^		
		*β*	95% CI	*p*	*β*	95% CI	*p*
Whole							
Aβ42/40	333	0.00	0.00,0.00	0.800	0.00	0.00,0.00	0.198
p-tau181	639	−0.12	−0.23,-0.01	0.040	−0.06	−0.18,0.06	0.312
NfL	638	−0.47	−0.58,-0.36	<0.001	−0.27	−0.38,-0.15	<0.001
A^−^							
Aβ42/40	183	0.00	0.00,0.00	0.339	0.00	0.00,0.00	0.296
p-tau181	295	−0.11	−0.18,-0.03	0.008	−0.09	−0.17,0.00	0.039
NfL	294	−0.47	−0.64,-0.29	<0.001	−0.28	−0.46,-0.09	0.004
A^+^							
Aβ42/40	150	0.00	0.00,0.00	0.414	0.00	0.00,0.00	0.318
p-tau181	344	−0.10	−0.29,0.09	0.296	−0.07	−0.27,0.14	0.521
NfL	344	−0.45	−0.58,-0.32	<0.001	−0.27	−0.40,-0.13	<0.001

^a^A^−^ represents Aβ-PET negative sample, A^+^ represents Aβ-PET positive sample. ^b^Adjusted for age and sex.

### 3.3. Renal function and the diagnostic performance of plasma biomarkers

**Figure 3:**
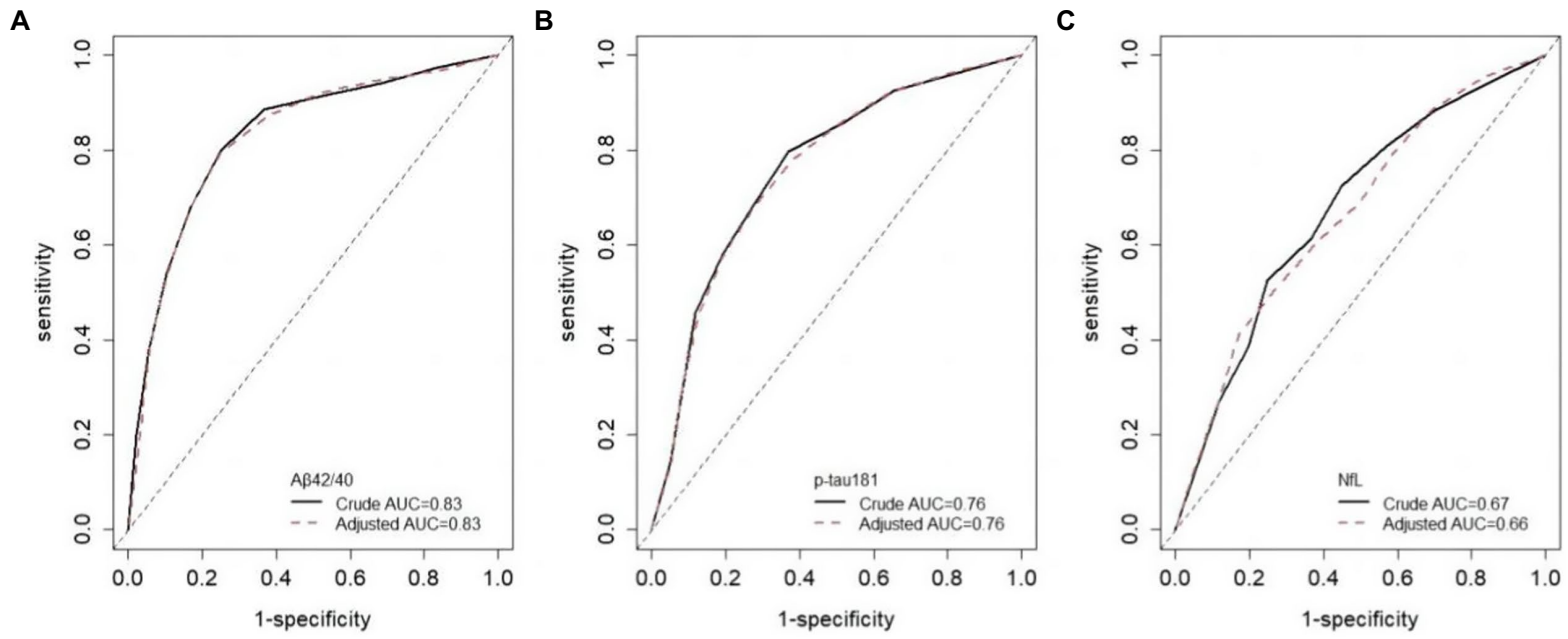
ROC curves adjusted for eGFR. Crude AUC means unadjusted AUC, adjusted AUC means AUC adjusted for eGFR.

Although there were slight differences in AUC for each biomarker between different eGFR groups, the results were not statistically significant (Table 3). The crude AUC and AUC adjusted for eGFR were nearly the same further supporting these results (Aβ42/40, 0.83 versus 0.83; p-tau 181, 0.76 versus 0.76; NfL, 0.67 versus 0.66; Figure 3). The cutoff values for Aβ42/40 in different eGFR groups did not differ. However, the cutoff for p-tau181 changed a lot in participants with eGFR 60–90 ml/min (15.15 pg./ml, a difference of 19%) and eGFR<60 ml/min (14.49 pg./ml, a difference of 23%) compared to participants with eGFR ≥90 ml/min (18.74 pg./ml). The cutoff for NfL changed little in participants with eGFR<60 ml/min (34.9 pg./ml, a difference of 4%) compared to participants with eGFR ≥90 ml/min (33.7 pg./ml). In contrast, the cutoff for NfL change a lot in participants with eGFR 60–90 ml/min (29.35 pg./ml, a difference of 13%) compared to participants with eGFR ≥90 ml/min (33.7 pg./ml). Overall, sensitivity, specificity, NPV, and PPV also changed with different cutoff values.

**Table 3:** Diagnostic performance of plasma biomarkers in participants with different renal function.

	N^a^ (N^+^, N^−^)	AUC (95% CI)	*p* value (compared with eGFR <60)	Cutoff^b^	Sensitivity	Specificity	PPV	NPV
Aβ42/40								
eGFR <60	54 (28,26)	0.80 (0.68–0.93)	–	0.12	0.93	0.65	0.74	0.89
eGFR 60–90	217 (100,117)	0.85 (0.79–0.90)	0.51	0.12	0.77	0.86	0.83	0.81
eGFR ≥90	62 (22,40)	0.78 (0.66–0.90)	0.782	0.12	0.91	0.63	0.57	0.93
p-tau181								
eGFR <60	111 (60,51)	0.80 (0.71–0.89)	–	14.49	0.88	0.63	0.74	0.82
eGFR 60–90	414 (226,188)	0.74 (0.69–0.79)	0.252	15.15	0.71	0.72	0.75	0.67
eGFR ≥90	114 (58,56)	0.82 (0.74–0.89)	0.778	18.74	0.62	0.89	0.86	0.69
NfL								
eGFR <60	110 (60,50)	0.69 (0.59–0.79)	–	34.9	0.87	0.48	0.67	0.75
eGFR 60–90	414 (226,188)	0.67 (0.61–0.72)	0.686	29.35	0.77	0.48	0.64	0.64
eGFR ≥90	114 (58,56)	0.72 (0.62–0.81)	0.691	33.7	0.48	0.89	0.82	0.63

^a^N represents whole sample, N^+^ represents Aβ-PET positive sample, N^−^ represents Aβ-PET negative sample. ^b^Cutoff values were calculated by Youden-Index.

## 4. Discussion

The main findings of this study were as follows. First, plasma Aβ42/40 ratio was not influenced by renal function, renal function was only found negatively associated with p-tau181 levels in A^−^ participants, while NfL levels increased with the decline of renal function. Second, renal function did not impact the diagnostic accuracy and cutpoint of Aβ42/40. Although the diagnostic accuracies of p-tau181 and NfL were not influenced by renal function, the cutpoints that were diagnosed for brain amyloid positivity differ depending on the levels of renal function.

Our results are in accordance with previous studies about the associations between renal function and plasma Aβ42/40 and NfL levels, which reported that eGFR was not associated with Aβ42/40 but negatively associated with NfL (Akamine et al., 2020; Pichet Binette et al., 2022). However, none of them investigated the impact of renal function on the diagnostic performance of Aβ42/40 and NfL. A previous study has reported the influence of CKD on the diagnostic performance of p-tau181(Mielke et al., 2022). They found participants with CKD had higher plasma p-tau181 levels and the exclusion of them affected the cutpoint of p-tau181, the results were similar to ours. However, CKD is a serious condition, which limited the generality of these findings. In our study, we included participants with mild to moderate renal function decline (lowest eGFR was 30.39 ml/min) and none of them had severe renal dysfunction. We found the cutoff values of p-tau181 were changed in participants with mild to moderate renal function decline compared to participants with normal renal function. Our results further highlight the effect of renal function on the reference ranges of p-tau181, even for people without CKD.

Animal studies have shown that kidney was involved in the peripheral clearance of Aβ, the abnormal renal function would raise the plasma levels of Aβ in mice (Xiang et al., 2015; Tian et al., 2021). Since renal dysfunction would rise plasma Aβ42 or Aβ40 alone (Syrjanen et al., 2022), these effects may offset leading to the Aβ42/40 ratio unchanged. Plasma p-tau181 and NfL levels increased with the decline of eGFR, which shows that the kidney may also be involved in the clearance of p-tau181 and NfL. Renal function decline could reduce the clearance of p-tau181 and NfL resulting in their plasma levels increasing. Only NfL levels were found to be affected by renal function in A^+^, but not for p-tau181. A possible explanation is that p-tau181 is a more specific biomarker for AD pathology compared with NfL (Chatterjee et al., 2022; Sarto et al., 2022), the impact of renal function on p-tau181 levels may be weaker in A^+^. This was consistent with our findings that the AUC and sensitivity of p-tau181 were higher than NfL. The cutoff values for p-tau181 and NfL were not raised with the decline of renal function in this study as expected, which may be due to the reduced sample size after being stratified into different eGFR subgroups. Our results need to be validated in future studies comprising larger sample sizes.

The strengths of this study were as follows. First, we examined the influence of renal function on the diagnostic performance of Aβ42/40 and NfL, this is the first time to our knowledge. It is crucial for the interpretation of results in the context of clinical use since plasma NfL levels were significantly influenced by renal function. Second, this study showed that even mild to moderate decline of renal function would influence the cutpoints of plasma p-tau181 and NfL. Our findings increased the generalizability of the influences of renal function on plasma biomarkers for AD. Third, we include participants with the time interval between index test and reference standard no more than 90 days, to avoid the disease progression bias. However, one limitation of this study is that most participants in ADNI were non-Hispanic White individuals who come from North America. The external validity of this study was limited and our results need to be validated in other regions comprising different racial and ethnic people. Second, creatinine was used to estimate eGFR in this study, more precise measures such as cystatin C could be used in the future. Third, many diseases were found to be associated with renal function and plasma biomarkers of AD such as diabetes and cardiovascular disease (Janelidze et al., 2016; Polymeris et al., 2020), the confounding effects of comorbidities were not examined in this study.

Taken together, plasma Aβ42/40 is a robust biomarker for AD with high diagnostic accuracies, which is less affected by renal function. Plasma levels of p-tau181 and NfL were affected by renal function, and the specific reference ranges of them need to be determined according to the renal function before clinical use.

## Data availability statement

The datasets presented in this study can be found in online repositories. The names of the repository/repositories and accession number(s) can be found at: adni.loni.usc.edu/.

## Ethics statement

The ADNI study was reviewed and approved by the local ethics committee. The patients/participants provided their written informed consent to participate in this study.

## Author contributions

BZ: study design, analysis of data, and manuscript drafting. CZ: study design and statistical analysis. YW and LC: revision of manuscript. YQ, YW, and DP: study design and revision of manuscript. All authors reviewed and approved the final version of the manuscript.

## Funding

This work was supported by the National Key R&D Program of China (grant no. 2022YFC2010103) and the Central health research project (grant no. 2020ZD10).

## Conflict of interest

The authors declare that the research was conducted in the absence of any commercial or financial relationships that could be construed as a potential conflict of interest.

## Publisher’s note

All claims expressed in this article are solely those of the authors and do not necessarily represent those of their affiliated organizations, or those of the publisher, the editors and the reviewers. Any product that may be evaluated in this article, or claim that may be made by its manufacturer, is not guaranteed or endorsed by the publisher.
